# Research of extraction behavior of heavy metal Cd in tea based on backpropagation neural network

**DOI:** 10.1002/fsn3.1392

**Published:** 2020-01-08

**Authors:** Chengli Guan, Yue Yang

**Affiliations:** ^1^ Yangjiang Polytechnic Yangjiang China; ^2^ Guangdong Provincial Key Laboratory of Atmospheric environment and Pollution Control South China University of Technology Guangzhou China

**Keywords:** health, leaching rate, prediction, safety

## Abstract

In order to meet the increasing demand for food and beverage safety and quality, this study focused on the application of a back propagation (BP) neural network to determine the leaching rate of heavy metal in tea to improve the scientific health of tea drinking. The evaluation index and target expectations have been determined based on the extraction experiment of heavy metal Cd in tea soaking, with 3 evaluation index values taken as input layer parameters and the heavy metal extraction rate taken as output layer parameter. Then, employ the sample data standardized by min‐max linearization method to train and test the network model and get the satisfactory results, which showed that the constructed BP neural network expressed a fast convergence speed and the systematic error was as low as 0.0003509. Additionally, there was no significance between Cd leaching rate of experimental results and neural network model results by reliability testing with a correlation coefficient was .9895. These results revealed that the network model established possessed an outstanding training accuracy and generalization performance, which effectively reflected the extraction rate of heavy metal in tea soaking and improved the safety of tea drinking.

## Graphical abstract

This work constituted the first attempt to simulate the leaching rate of heavy metal for tea soaking using a BP neural network. It demonstrated that a BP neural network is effective for predicting the safety evaluation research for tea drinking with a reduced number of trial‐and‐error experimental cycles and provides useful guidance for the development of tea industry and food industry.

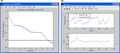

## 1 INTRODUCTION

Tea is one of the most popular nonalcoholic beverages in the world (Cabrera, Gimenez, & Lopez, 2003). It is also a traditional healthy drink with resource advantages and cultural heritage in China (Ma, Wan, & Huang, 2015). Tea contains a variety of inorganic mineral elements and organic compounds, including sodium, phosphorus, calcium, copper, cadmium, amino acids, tea polyphenols, vitamins, alkaloids, and leaf proteins (Cabrera et al., 2003; Tanmoy & Bhagat, 2010). So tea can not only reduce the risk of cardiovascular disease and lower cholesterol, its trace elements are also a beneficial supplement to the human body (Chung, Schwartz, & Herzog, 2003; Fernandez, Pablos, & Martin, 2002). However, tea is generally consumed after boiling water or hot water. Some of the ingredients contained in the tea will enter the body through tea soup, including heavy metal elements such as copper, lead, and cadmium, which will bring certain potential harm to human health (Lv, Lin, & Tan, 2013; Ma et al., 2015). With the increasing awareness of environmental protection and the increasing demand for safe food, the quality and safety of tea, especially the heavy metal residues that have an important impact on human health, have attracted the attention of scholars worldwide (Matsuura, Hokura, & Katsuki, 2001; Seenivasan, Manikandan, & Muraleedharan, 2008; Shen & Chen, 2008; Zhang et al., 2011; Zhou & Li, 2008). In addition, Ma et al. (2015) studied the health risk levels through the ingestion pathway of five heavy metals in tea, namely Mn, Cu, Zn, Pb, and Cd, in which Cd had the greatest impact and the health risk value was close to the limit. Therefore, it is of great value and practical significance to study and evaluate the leaching rate of heavy metal Cd during tea soaking.

Actually, the leaching amount and leaching rate of heavy metals in tea are related to many factors such as tea type, brewing temperature, and brewing frequency (Ma et al., 2015). Moreover, the dissolution of heavy metals in tea is a dynamic process, and there are many factors that affect this process. The influence of each element is of different degree and cannot be studied simply using linear evaluation methods such as fuzzy comprehensive evaluation method (Ying & Liu, 2015). So we have to do the complicated single experiment to find the optimal value firstly and then through a large number of orthogonal experiment to seek optimal values under the influence of many factors. This method is too complicated to daily application.

These works indicate that a promising method which requires less effort and labor is needed for the research of heavy metals in tea soaking. And the information science approach as a nonlinear simulation technology, that is an artificial neural network (ANN) (Fan, He, & Lin, 2014; Hattori & Kito, 1995; Yang, Yu, & Guan, 2018) including Hopfield neural network, adaptive resonance technology, fuzzy neural network, and backpropagation (BP) neural network, is structured to study the relationship between a set of factors and the results. According to research of Wang et al. (2013), BP neural network is most widely used in various evaluation fields (Chen, 2014; Ding & Jia, 2012; Fan, Piao, & Chen, 2012; Ju, Tade, & Zhu, 2004) for its good performance of self‐learning, self‐adapting, self‐organizing, and self‐reasoning (Maier, Jain, & Dandy, 2010; Miao, Zhao, & Gao, 2010; Palani, Liong, & Tkalich, 2008), which was proposed by Rumelhart, Hintont, and Williams (1986). Yang et al. (2018) applied BP neural network to the performance evaluation of environmental catalytic materials and pollutant purification research; Fan et al. (2014) applied BP neural network to the corrosion prediction research of petrochemical tower; Ju et al. (2004) applied BP neural network to the prediction of hydrogen content in coal resources, etc.

In this reported work, in order to better understand the extraction behavior of heavy metals in tea soaking, the leaching rate of heavy metal Cd was studied and simulated with a BP neural network model established on the basis of the experimental data. To the best of our knowledge, this is the first trial to predict the safety performance of heavy metals for tea under different process conditions through the BP neural network. This study could offer a new path for the investigation of tea brewing, and the results could be useful for guiding future experimentation and application processes of drinking quality safety to produce the desired effect.

## 2 MATERIALS AND METHODS

### 2.1 Chemicals

All the chemical reagents used in the experiments were of analytical grade. The tea used in the study was green tea with food production license, which was purchased from the local market of Yangjiang City, Guangdong Province. Cadmium chloride was used to make the standard working curve of heavy metal Cd. Nitric acid and hydrogen peroxide are used as auxiliary reagents for microwave digestion of tea. The fresh ultrapure water was used to prepare all solutions.

### 2.2 Experimental method

#### 2.2.1 Measurement of content of heavy metal Cd

First, the stratified sampling method was used to divide the purchased tea into 3 layers. About 1.00 g of tea randomly weighed in each layer, which was dried at 105℃ for 2 hr and with constant weight, was added to a microwave digestion tank with a capacity of 100 ml for microwave digestion by mixing in 5.0 ml HNO_3_ and 2.0 ml H_2_O_2_. Along with digestion, the pressure was stable at 1.5 MPa and the digestion time was 20 min. After that, the absorbance of heavy metal Cd in the digested liquid was determined by flame atomic absorption spectrophotometry at a measurement wavelength of 228.8 nm, a lamp current of 3.0 mA, and a burner height of 6.5 mm (Shi, Ma, & Han, 2008). And the concentration of Cd was calculated according to the standard working curve of heavy metal Cd. The content of heavy metal Cd was calculated with the following equation:

$$m_{1}=\;c\;\;\times \;V$$

where *m*_1_ (mg) is the content of heavy metal Cd in the tea added, *c* (mg/L) and *V* (L) are the concentration of Cd in the digestion solution and the solution volume, respectively. Moreover, to ensure data reliability, three independent parallel experiments were performed each time and the arithmetic mean was taken.

#### 2.2.2 Measurement of leaching rate of heavy metal Cd

According to the public's habits and behaviors of tea drinking, the batch leaching rate experiments about the effects of the number of tea soaking (from 1 to 3), soaking time (for 1, 5, 10, 20, 30, 60, and 120 min, respectively), and soaking temperature (at 50, 60, 70, 80, 90, and 100℃, respectively) on the leaching rate of heavy metal Cd were conducted. First, add 1.00 g of tea sample to a series of 100 ml beakers, respectively, followed by the addition of 50 ml fresh ultrapure water. Then, place the beakers in a water bath at the temperature from 50 to 100℃ for the time from 1 to 120 min. Finally, the prepared supernatant solution after centrifugation at 5,000 r/min for 5 min was taken to measure the content of heavy metal Cd. To determine the effect of the number of the tea soaking on the leaching rate of Cd, the primary filter residue obtained from the first immersion would be soaked in accordance with the above conditions, and the second immersion and filter residue were produced. By analogy, the third leaching solution was obtained. The total amount of leaching is the sum of the leaching amount of three times. And based on the three influencing factors, 32 sets of experimental data were obtained. The leaching rate of heavy metal Cd can be expressed as below:

$$\eta \;=\;\left(m_{2}/m_{1}\right)\;\times 100\%$$

where m_2_ (mg) was the content of heavy metal Cd in soaking solution.

### 2.3 Prediction method of neural network

**Figure 1:**
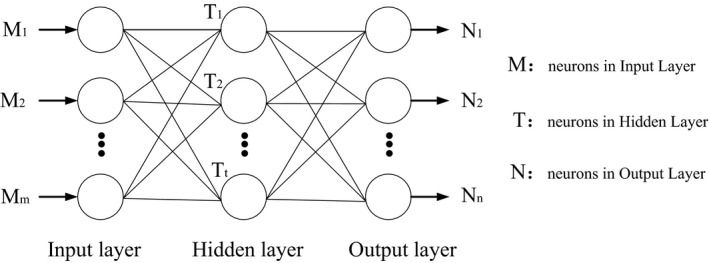
Schematic diagram of the topological structure of the neural network

The error backpropagation (BP) feedforward network is a typical feedforward network and is currently the most widely used. The information flow of the feedforward network is transmitted from the input layer to the lower layer, and there is no feedback information flow. After the network processing, the target signal is gained from the output layer. And in the multi‐layer feedforward network, the input and output neurons are connected with the outside world and directly feel the external stimulus. While the middle layer has no direct connection with the outside world, it is called the hidden layer. Since multiple hidden layers were added to the multi‐layer feedforward network in the structure, the ability of artificial neural network to process complex information is greatly improved (Ding, Su, & Yu, 2011). Through training, the multi‐layer network can realize the nonlinear mapping of M → N due to the introduction of hidden layer and nonlinear transfer function. A schematic diagram of the topological structure of the neural network is shown as Figure 1. Just because the mapping of any nonlinear function from input layer to output layer can be obtained by choosing appropriate network structure and hidden layers, a BP neural network model is established to predict and analyze the leaching rate of heavy metal Cd in tea by using MATLAB 7.1 software combined with experimental analysis in this paper. And the running environment is Windows XP system, and the processor is Intel Core i5 with 4.00 GB RAM.

Suppose the input layer is *M* with m neurons, the hidden layer is *T* with *t* inner neurons, and the output layer is *N* with *n* neurons, the transfer function of the hidden layer is *f*_1_, the corresponding activation function of output layer is *f*_2_, the output of the network is *A*, and the desired output is *S*. Then, the forward propagation process of the information is as follows:

Output in the hidden layer: $a_{1i}=f_{i}\left(\sum_{j=1}^{m}w_{1ij}M_{j}+b_{1i}\right),\;i=1,2,\ldots ,t$

Output in the output layer: $a_{2k}=f_{2}\left(\sum_{i=1}^{t}W_{2ki}a_{1i}+b_{2k}\right),\;k=1,2,\ldots ,n$

The error function is defined as: $E\left(W,B\right)=\frac{1}{2}\sum_{k=1}^{n}{\left(s_{k}-a_{2k}\right)}^{2}$

In addition, the back propagation of the error and the process of weight change are as follows: the weight change of the output layer from the *i*‐th input to the *k*‐th output:

$$\Delta w_{2ki}=-\eta \frac{\partial E}{\partial w_{2ki}}=-\eta \frac{\partial E}{\partial a_{2k}}\frac{\partial a_{2k}}{\partial w_{2ki}}=\eta \left(s_{k}-a_{2k}\right)f_{2}^{\prime }a_{1i}=\eta \delta_{\mathrm{ki}}a_{1i}$$

where, $\delta_{\mathrm{ki}}=\left(s_{k}-a_{2k}\right)=e_{k}f_{2}^{\prime },\;e_{k}=s_{k}-a_{2k}$

The weight change of the hidden layer from the *j*‐th input to the *i*‐th output is as follows:

$$\begin{array}{c} \Delta w_{1ij}=-\eta \frac{\partial E}{\partial w_{1ij}}=-\eta \frac{\partial E}{\partial a_{2k}}\frac{\partial a_{2k}}{\partial a_{1i}}\frac{\partial a_{1i}}{\partial w_{1ij}}\\ \;\;\;\;\;\;\;\;=\eta \sum_{k=1}^{n}\left(s_{k}-a_{2k}\right)\;f_{2}^{\prime }w_{2ki}f_{1}^{\prime }M_{j}\\ \;\;\;\;\;\;\;=\eta \delta_{\mathrm{ij}}M_{j} \end{array}$$

$$\text{where}\;\delta_{\mathrm{ij}}=e_{i}f_{1}^{\prime },\;e_{i}=\sum_{k=1}^{n}\delta_{\mathrm{ki}}W_{2ki},\;\Delta b_{1i}=\eta \delta_{\mathrm{ij}}$$

## 3 RESULTS AND DISCUSSION

### 3.1 Preparation of sample data

Shanker, Hu, and Hung (1996) studied the influence of data standardization on network training and proved that it has a good effect on the running of the neural network model. Generally, the method of data normalization is to transform the original data into the range of [0, 1] in order to improve the accuracy of the model, and so it is convenient for data simulation. In this work, for the sake of limiting the input and output range of sample data, all data are converted to the range of [0.1, 1] by linearization method. Therefore, there would be no data overflow or no data into the activation range with a very small slope. The linear form is given by:

$$m_{i}=0.1+0.9\times \left[\left(x_{i}-x_{\min}\right)/\left(x_{\max}-x_{\min}\right)\right].$$

where *x*_min_ and *x*_max_ are the minimum and maximum value of the sample data, respectively; *x*_i_ is the sample data before conversion, and *m*_i_ is the corresponding converted data. According to the experiments of the leaching rate of heavy metal, 32 groups of data about the leaching rate of heavy metal Cd in green tea were obtained. And the standardized sample data are shown in Table 1.

**Table 1:** The training and prediction samples

No.	Number of tea soaking	Time of tea soaking/min	Temperature of tea soaking/K	Leaching rate of Cd/%
1	0.55	0.16807	1.0	0.74367
2	1.0	0.16807	1.0	0.84051
3	0.10	0.13025	1.0	0.24241
4	0.10	0.16807	0.82	0.41329
5	0.55	0.31933	1.0	0.81203
…	…	…	…	…
28	0.10	0.31933	1.0	0.55569
29	0.55	0.54622	1.0	0.85760
30	1.0	0.31933	1.0	0.91456
31	0.55	0.16807	0.46	0.33354
32	1.0	0.16807	0.46	0.38481

### 3.2 Establishment of network model

The neural network is a parallel and distributed information processing network (Li, Zeng, & Shui, 2008). The network structure is composed of many neurons in general. Each neuron has a single output, which can be connected to many other neurons. The input of the network has multiple connection paths, and each connection path corresponds to a connection weight coefficient. The structure of BP neural network system directly affects its function mapping ability, while the number of hidden layers and the number of neurons in each hidden layer are crucial factors. And the number of hidden layers of the neural network can be infinite in theory. However, in actual construction, too many hidden layers will bring a lot of calculation, then increase the complexity of the network and affect the training time. According to the existence theorem of mapping: “given any continuous function *f*:U^n^ → R^m^, *f*(*X*) = *Y*. Here *U* is a closed unit interval [0, 1], f can be accurately implemented with a three‐layer perceptron network” (Guan, Yang, & Chen, 2015; Yang, Guan, & Chen, 2014). The research shows that if we want to achieve any nonlinear mapping relationship of the single hidden layer neural network system under the predetermined accuracy, we can choose the S‐type transfer function for the hidden layer and the linear function for the output layer (Liu, Tang, & Jin, 2008). Consequently, a single hidden layer in the network model was set up in this study.

### 3.3 Determination of the number of neurons

**Figure 2:**
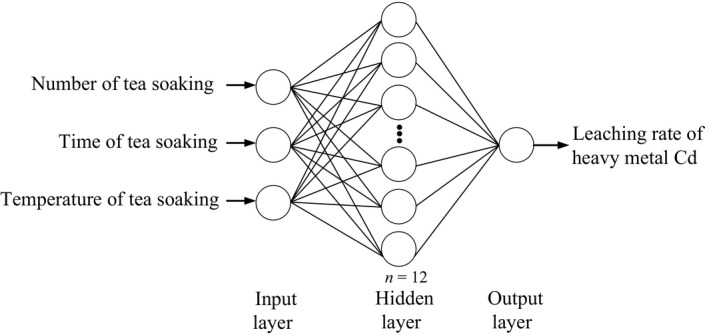
Sketch map of BP neural network

The number of input and output neurons refers to the number of input and output variables, which are all related to the sample data. The network model of heavy metal leaching rate is to establish the functional relationship between the leaching rate of Cd and the number of tea soaking, time and temperature. Hence, the network model established in this work is a three‐input & single‐output structure, where the number of input neurons is 3 and the number of output neurons is 1. In addition, the number of neurons in the hidden layer directly affects the expression ability of the network. Too few neurons will not be sufficient to express the complex nonlinear relationship of the system, while too many neurons will lead to over‐fitting and result in the decline of the generalization ability of the network model. Considering the network accuracy and the generalization performance, the number of neurons in the hidden layer is determined based on the number of neurons in the input and output layers by the following equation (Yin, Wang, & Yan, 2011):

$$t=\sqrt{m+n}+i,\;0\leq i\leq 10.$$

in which, *t*, *m*, and *n* are the number of neurons in the hidden layer, input layer, and output layer, respectively, and *i* is the number of the sample data for the training of network, taken to 10 in this case. On the basis of the formula, *t* = 12 is obtained in combination with the model running. Accordingly, the constructed BP neural network model structure is shown in Figure 2.

### 3.4 Operating platform of BP neural network

The prediction system of BP neural network is designed by using the graphical user interfaces (GUI) provided by MATLAB. For this reason, the users can employ the neural network toolbox of MATLAB more simply and conveniently. Almost all the command can be accomplished graphically in the GUI such as data input and output, network establishment, initialization, training, and simulation. It can not only avoid the relatively complicated and intuitive command program, but also reduce the time of program editing and debugging, and improve the efficiency and quality of problem‐solving. The prediction system constructed in this paper has good performance for model training and predicting. And the “training” button is taken as an example to illustrate the implementation of the button control function:



function pushbutton_xunlian_Callback(hObject, eventdata, handles);
global net;
xunlian_yangben = str2num(get(handles.edit_xunlian_yangben, 'String'));
xunlian_yangben = xunlian_yangben';
size_xunlian_yangben = size(xunlian_yangben); if(size_xunlian_yangben([1])==get(handles.popupmenu_shuruceng_num, 'Value')).
xunlianjieguo = sim(net,xunlian_yangben);
xunlianjieguo = xunlianjieguo';
set(handles.pushbutton_baocun, 'Enable','on');
else;
warndlg;
end.




### 3.5 Training and predicting process

**Figure 3:**
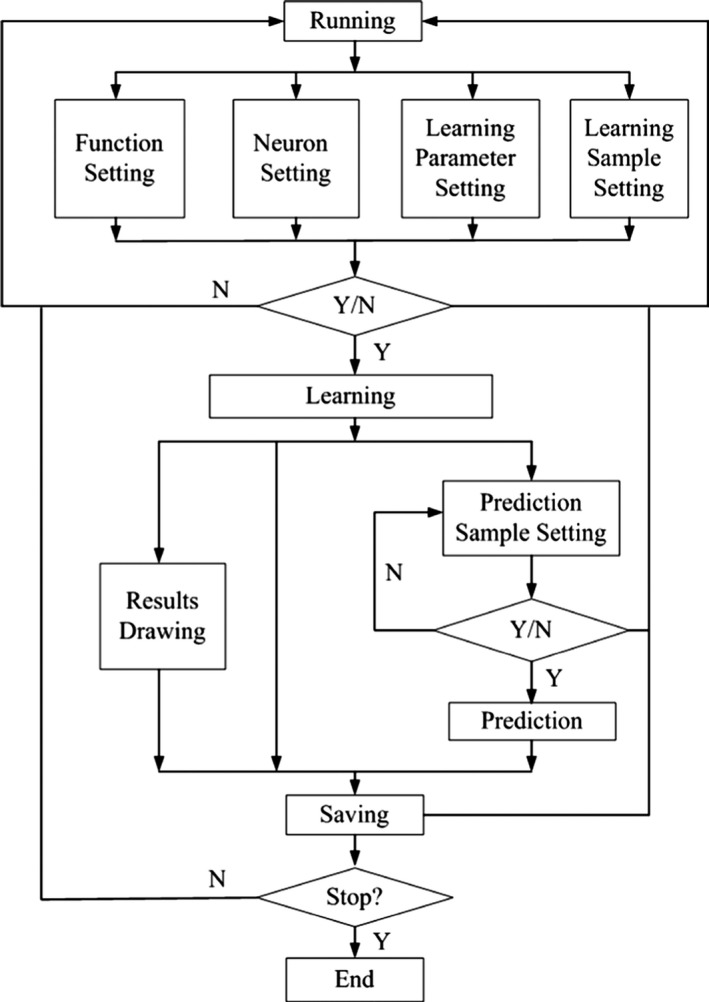
Operation process of the BP neural network

The BP neural network model is trained and simulated on the grounds of the flow chart shown in Figure 3. Operating the model, the main interface of the GUI appears. And the first work is to set up the parameters of the BP neural network. In conformity with the practice, “tansig” is used as the transfer function of hidden layer, “pureline” as the transfer function of output layer, “trainlm” as the training function, “learngd” as the learning function, and “mse” as the performance function in this model, respectively. Besides, there is another parameter affecting the applicability of the model, that is the training accuracy which is the target value of the error performance of the network training. If it is too small, the network fits the training set well, but the training time is too long to reach the prediction result; while if it is too large, it cannot approximate the training set very well, let alone predict. So through the comparison, the training accuracy is taken to 0.001 in this work to ensure that the relative error between training set and prediction set is close.

**Figure 4:**
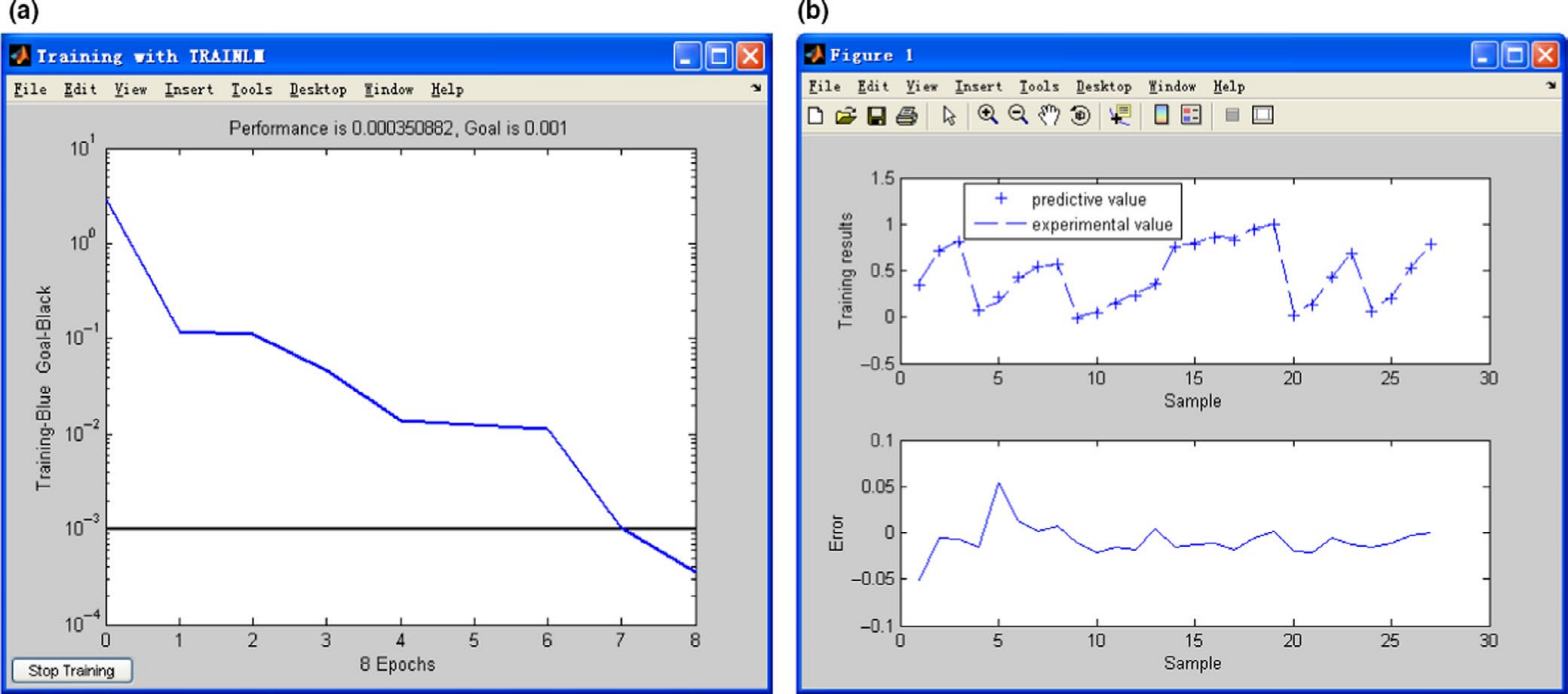
Training process (a) and training result (b) of BP neural network

Second, make use of the known sample data to train the network model. When the training of BP neural network is accomplished, the curve of the training process is presented. And press the button of “Draw Training Results,” a training result graph will pop up. It is worth noting that when training or predicting is carried out, a warning dialog box will appear when there is a disagreement between the setting of BP neural network and the sample data input. In this case, the 27 groups of sample data randomly chosen in Table 1 were taken to train the neural network with an upper limit of the number of iterations as 1,000. Through running the model, the graphs of training process and training results were produced as shown in Figure 4. According to the Figure 4a, the BP neural network model of leaching rate of heavy metal Cd in tea has a fast convergence speed. By only eight iterations of training, the systematic error has reached 0.0003509 which achieves the reserved requirement for training accuracy. And from the Figure 4b, the actual output of the network is close to the target output. And among the 27 groups of training sample data, there are 26 groups of samples with a mean square error which is less than 5.0%. The results revealed that the BP neural network model established can preferably approximate the regulation of leaching rate of heavy metal Cd for tea soaking by the training sample data.

**Figure 5:**
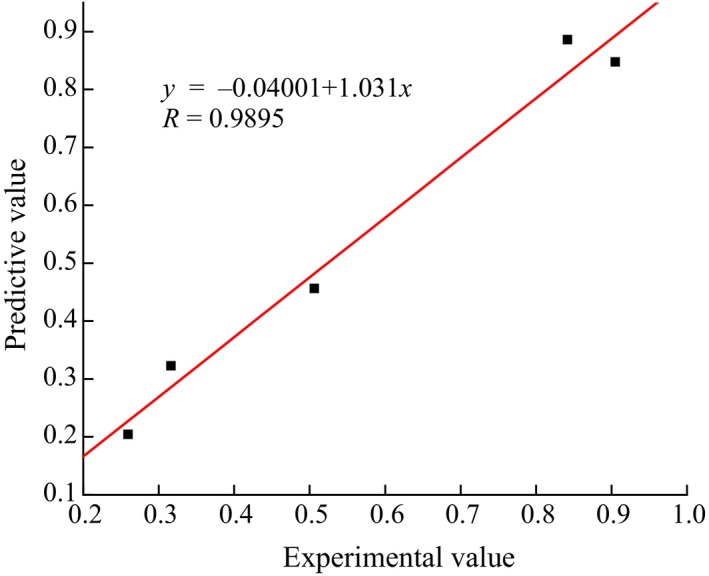
Comparison between predictive values from the BP neural network and experimental values

Finally, the trained BP neural network can be used to predict the unknown samples. Running the network, the remaining 5 test samples in Table 1 were output to the model in turn, and the predicted results were compared with the experimental values as displayed in Figure 5. It can be seen from the results that the predicted value comes very close to the experimental value with the maximum absolute error as 0.0576. While the error itself is inevitable, so long as it is within the allowable range, it can be considered effective. Furthermore, the correlation coefficient between the predicted results and the experimental results is 0.9895, which suggested that the neural network constructed has great generalization performance, and the fitting of the nonlinear mapping relationship between the model output and the ideal output is accurate.

## 4 CONCLUSIONS

In this reported work, we successfully established a BP neural network based on MATLAB software and applied the network to conduct the exploratory research on the leaching rate of heavy metal Cd for tea soaking. The results of network operation showed that the systematic error reached the predetermined requirement of training accuracy as low as 0.0003509, and the correlation coefficient of the simulated data and experimental data was 0.9895, which indicated that the BP neural network expressed the capability to exactly simulate the experimental results.

This work carried out the first attempt to predict the leaching rate of heavy metal for tea soaking under different brewing conditions by employing a BP neural network. And we utilized the network model to find out the optimal process conditions of tea soaking overcoming the complicated and nonlinear influence of evaluation factors. It demonstrated that the BP neural network is scientific and objective for simulating the safety evaluation research for tea drinking by judging the leaching rate of heavy metal accurately with a reduced number of trial‐and‐error experimental cycles. Furthermore, it provides effective suggestions for the development of tea industry and food industry.

## CONFLICT OF INTEREST

The authors declare that they do not have any conflict of interest.

## ETHICAL APPROVAL

This study does not involve any human or animal testing.
